# A Review and Meta-Analysis of Age-Based Stereotype Threat: Negative Stereotypes, Not Facts, Do the Damage

**DOI:** 10.1037/a0038586

**Published:** 2015-01-26

**Authors:** Ruth A. Lamont, Hannah J. Swift, Dominic Abrams

**Affiliations:** 1 Centre for the Study of Group Processes, University of Kent

**Keywords:** stereotype threat, age, performance, cognition, memory

## Abstract

Stereotype threat effects arise when an individual feels at risk of confirming a negative stereotype about their group and consequently underperforms on stereotype relevant tasks (Steele, 2010). Among older people, underperformance across cognitive and physical tasks is hypothesized to result from age-based stereotype threat (ABST) because of negative age-stereotypes regarding older adults’ competence. The present review and meta-analyses examine 22 published and 10 unpublished articles, including 82 effect sizes (*N* = 3882) investigating ABST on older people’s (*M*_age_ = 69.5) performance. The analysis revealed a significant small-to-medium effect of ABST (*d* = .28) and important moderators of the effect size. Specifically, older adults are more vulnerable to ABST when (a) stereotype-based rather than fact-based manipulations are used (*d* = .52); (b) when performance is tested using cognitive measures (*d* = .36); and (c) occurs reliably when the dependent variable is measured proximally to the manipulation. The review raises important theoretical and methodological issues, and areas for future research.

Older adults face pervasive negative stereotypes that memory, cognitive, and physical competence decline with age (Cuddy & Fiske, 2002). These stereotypes make them vulnerable to age prejudice, discrimination, and age-based stereotype threat (ABST). Stereotype threat arises when an individual faces a situation that puts them at risk of confirming a negative stereotype about their group. As Steele (2010, p. 59) describes: “They know at some level, that they are in a predicament: Their performance could confirm a bad view of their group and of themselves, as members of that group” (p. 59). This threat results in underperformance on stereotype relevant tasks (Steele & Aronson, 1995).

There is now more than a decade of research accumulating to show that older adults may be vulnerable to ABST when they perform memory, cognitive, or physical tasks (Abrams, Crisp, Marques, Fagg, Bedford, & Provias, 2008; Hess, Auman, Colcombe, & Rahhal, 2003; Swift, Lamont, & Abrams, 2012). These effects have important social and economic implications, which are particularly relevant given population aging and aging workforces. However, not all older adults are vulnerable to ABST effects (e.g., Andreoletti & Lachman, 2004; Fritzsche, DeRouin, & Salas, 2009). This raises important empirical questions about the size and scope of ABST effects and the circumstances under which older adults are more or less vulnerable to ABST, which we investigate in this article.

Stereotype threat is theorized to operate through distinct motivation-based mechanisms, often linked to emotion (distinct from automatic or “cold” priming effects, see Wheeler & Petty, 2001). It is this experienced threat that is said to undermine performance and lead to behavioral confirmation of a stereotype. Steele’s (2010) account of stereotype threat highlights some important elements. First, although they do not necessarily have to endorse the stereotype, individuals must recognize that they belong to the stereotyped group and be mindful of the stigma attached to that social group—the stereotype must be self-relevant (Steele & Aronson, 1995). Second, stereotype threat is a fluid, situational threat—the situation must present a risk of confirming the stereotype. These factors present a threat to one’s identity due to the value that is placed on having a positive and distinct social identity, as highlighted by social identity theory (Abrams & Hogg, 1990; Tajfel & Turner, 1979).

## Group-Specificity of Stereotype Threat

Stereotype threat effects have been studied across different negatively stereotyped social groups (e.g., women, gay men, ethnic minorities; Major, Spencer, Schmader, Wolfe, & Crocker, 1998; Steele, James, & Barnett, 2002; von Hippel et al., 2005). Three previous meta-analyses examined stereotype threat among ethnic minority groups and among women (Nadler & Clarke, 2011; Nguyen & Ryan, 2008; Walton & Spencer, 2009). Walton and Spencer (2009) found similarly sized stereotype threat effects affecting ethnic minorities and women, whereas Nguyen and Ryan (2008) found stronger effects for race/ethnicity (*d* = .32) than gender (*d* = .21). Importantly, Nguyen and Ryan (2008) and Shapiro (2011) highlight the diversity in the experience of stereotype threat associated with different group memberships. Given that there are some quite distinctive features of age prejudice and stereotypes, we cannot assume that the size and relevant predictors of ABST are the same as stereotype threat effects for other groups (Shapiro, 2011).

In contrast to gender and race/ethnic groups, age boundaries defining old age are construed more fluidly. This potentially makes the application of age stereotypes and stereotype threat a more subjective and variable experience. Aging stereotypes also have the potential to become relevant for everyone as people learn and internalize negative age stereotypes when they are young (Levy & Banaji, 2002), which then become self-relevant as people reach old age. Moreover, becoming “old” applies to the *majority* of the population, meaning the potential social, psychological, and economic impact of ABST is substantial. Thus, we considered that a review of the size and scope of ABST is both a necessary and timely contribution to the literature.

## Review of Age-Based Stereotype Threat Literature

At the time of writing, 22 published articles have tested ABST effects, several of which failed to find effects on performance (Andreoletti & Lachman, 2004; Hess & Hinson, 2006; Hess, Hinson, & Hodges, 2009; Horton, Baker, Pearce, & Deakin, 2010; Kang & Chasteen, 2009; O’Brien & Hummert, 2006). Others found that ABST effects were dependent on situational and individual factors such as, prevention or promotion focus (Gaillard, Desmette, & Keller, 2011), or the level of task demands (Hess, Emery, & Queen, 2009). Moreover, some studies have shown that ABST manipulations can improve performance (Fritzsche et al., 2009). A meta-analysis exploring effects of explicit positive versus negative age primes revealed a significant effect on memory performance (*d* = 0.38; Horton, Baker, Pearce, & Deakin, 2008). Although they are indicative, we cannot directly extrapolate these findings to ABST because explicit primes (e.g., a sentence unscrambling task) do not necessarily meet the criteria for ABST.

The mixed findings across ABST studies highlight the need for a review and meta-analysis to understand what factors might moderate ABST effects. Therefore, as well as focusing directly on ABST, the present meta-analysis complements Horton, Baker, Pearce, and Deakin (2008) in several ways. It includes the much larger set of articles and effect sizes available to date and also includes unpublished studies to examine potential publication bias. Moreover, because the literature highlights a number of potential moderators of ABST, the present meta-analysis examines conditions under which ABST effects flourish or diminish and the impact of ABST in different performance domains and types of population.

### Experimental Differences

#### Experimental manipulations of ABST

A variety of manipulations have been used to test ABST. Some manipulations explicitly state negative expectations regarding aging whereas others subtly reference the relevance of the task to age stereotypes. Previous meta-analyses of stereotype threat effects have compared explicit/implicit or blatant/subtle stereotype threat manipulations (Nadler & Clarke, 2011; Nguyen & Ryan, 2008). However, we propose that stereotype threat manipulations can be categorized more clearly based on their factual content versus allusion to a stereotype, which we label *fact-based* and *stereotype-based* manipulations, respectively.

#### Fact-based

In many studies ABST is manipulated by presenting factual statements of age-based differences to affect participants’ expectations about performance. For example, O’Brien and Hummert (2006, p. 347) told those in the threat condition that “past research has shown that memory performance declines with age.” For a researcher to present evidence that supports age-differences in performance is arguably going beyond “stereotype” threat.

#### Stereotype-based

Other studies have used stereotype-based manipulations. For example, Abrams, Eller, and Bryant (2006, p. 694) stated that “it is widely assumed that intellectual performance declines with age.” Other stereotype-based manipulations rely on more subtle cues—such as an age comparison or framing the task as stereotype-relevant—to activate negative stereotypes of aging (e.g., Chasteen, Bhattacharyya, Horhota, Tam, & Hasher, 2005; Desrichard & Kopetz, 2005; Swift et al., 2012).

Stereotype-based manipulations could be considered a purer form of stereotype threat manipulation, whereby the threat comes solely from societal stereotypes (Steele & Aronson, 1995). In contrast, presenting facts about age-based differences in competence not only acts as a reminder of societal stereotypes, but also gives credibility and removes ambiguity surrounding these stereotypes. Therefore, the distinction between operationalizations of fact-based and stereotype-based stereotype threat manipulations allows us to more clearly explore the impact that stereotyping has on older adults.

Stereotype-based cues could be more of a threat to performance outcomes because they introduce greater ambiguity in a performance situation. Although the “subtlety” of stereotype-based manipulations varies, they are overall more ambiguous than fact-based manipulations due to their omission of this factual evidence, and therefore may have a greater negative impact on older adults. Ambiguity and uncertainty about the application of the negative age stereotype may increase distracting thoughts (Hirsh, Mar, & Peterson, 2012), which in turn deplete cognitive resources needed for the task at hand (Schmader, Johns, & Forbes, 2008). Indeed, when testing the distinction between “implicit” and “explicit” stereotype cues, a number of studies have actually contrasted fact- and stereotype-based manipulations. For example, Kray, Thompson, and Galinsky (2001, Study 3) found that fact-based cues (defined as blatant) led participants to behave contrary to expectations defined by the stereotype, in line with “stereotype reactance” theory (Brehm, 1966); whereas stereotype-based cues (defined as subtle) led to behavioral assimilation, as predicted by stereotype threat theory (Steele & Aronson, 1995).

#### Stereotyped performance domains

ABST effects have been investigated in a number of performance domains including tests of memory, cognitive and physical ability, skill acquisition, and driving. ABST may vary according to performance domain if different skill-sets and resources are required for these different types of tasks (Beilock, Jellison, Rydell, McConnell, & Carr, 2006; Schmader et al., 2008). According to Schmader, Johns, and Forbes’ (2008) model of stereotype threat processes, stereotype threat might affect controlled processing due to heightened physiological response, increased task monitoring, and attempts to suppress negative emotion. All of these can cause cognitive depletion, reducing working memory capacity and the ability to perform tasks requiring controlled processing.

It is less clear how stereotype threat affects motor skills, which are less dependent on cognitive resources and controlled processing, and more reliant on unconscious or automatic processing. However, some research suggests that stereotype threat may affect physical performance if the individual attends too much to largely proceduralized tasks (Baumeister, 1984; Beilock et al., 2006), or if threatened individuals alter performance goals (Smith, 2004; Stone & McWhinnie, 2008). At present, ABST effects on motor skills have produced mixed findings (Horton et al., 2010; Swift et al., 2012).

Differences in ABST effects between performance domains may also suggest that the different content of aging stereotypes poses different levels of threat to older adults. Some research has suggested that the extent to which individuals identify with the performance domain and see it as important can moderate ABST effects, such that ABST effects are stronger on domains that are highly valued (Hess et al., 2003; Joanisse, Gagnon, & Voloaca, 2013). Although too few studies have measured domain identification to test it as a moderator in this meta-analysis, stronger ABST effects on one performance domain over another may indicate the relative strength of the particular aging stereotype and the subsequent increased vulnerability of older adults on that particular performance domain. The present meta-analysis therefore examines differences in ABST effects in several performance domains.

#### Baseline conditions

Across the ABST literature we discerned two types of baseline conditions. *Control* baseline conditions do not mention the age/stereotype relevance of the task, whereas *nullification* baseline conditions attempt to challenge the relevant negative age stereotype. Comparing the use of control versus nullification conditions provides useful insights for reducing the impact of ABST effects. For instance, in situations that may present an ABST, it is important to know whether it is better to avoid all mention of age (as with control conditions) or to present counterarguments to commonly held stereotypes (stereotype nullification). This has implications for the subtlety of campaigns that aim to encourage counterstereotypical behaviors such as active aging campaigns or advertising for later-life learning.

### Sample Characteristics

Previous stereotype threat meta-analyses have not investigated the extent to which sample characteristics moderate stereotype threat effects (Horton et al., 2008; Nadler & Clarke, 2011; Nguyen & Ryan, 2008; Walton & Spencer, 2009). Understanding effects of sample characteristics helps to provide parameters for the generalizability of findings. In the present analysis we focus on demographic details available across most studies. These are the age, gender, and the region in which research participants live.

#### Age

Given the arbitrary category boundaries that define “young” and “old,” the age of participants used in ABST studies could plausibly affect the strength of ABST. It might be expected that ABST has a greater effect on older people, due to the increased self-relevance of aging stereotypes. However, Hess, Emery, and Queen (2009) noted a greater effect on the performance of those age 60–70 years than those age 71–82 years. Similarly, Hess and Hinson (2006) found threat-based effects were most evident around the age of 68, but not evident among the older participants. It is suggested that those entering “old age” find the implications of this category membership more salient due to its relative newness; this creates a heightened sense of self-relevance (Hess et al., 2009). We therefore test whether the mean age of the study sample moderates ABST effects.

#### Gender

Stereotypes of aging have been argued to be more self-relevant for women (Levy, Ng, Myers, & Marottoli, 2013). There are notions of a “double-standard” of aging whereby it is less acceptable for women to show the signs of aging than for men (Sontag, 1997). Stereotype threat research has further suggested that multiple social identities can be involved in performance situations. For instance, alternative positive social identities may act as a buffer against stereotype threat (e.g., Rydell, McConnell, & Beilock, 2009). However, additional stigmatized social identities, such as being older and a woman, could magnify stereotype threat. If these ideas are correct it can be predicted that studies including a higher proportion of women should also display larger effects of ABST.

#### Region of study

The reviewed studies were carried out in a number of countries (United States, Canada, England, France, Belgium, and Romania). There may well be macrosocial contextual moderators of ABST. These might range from the transitory salience of specific very old people (e.g., the Queen) to more stable differences between cultures, including the age profile of the population and the role and status of older adults within that culture. There are cross-cultural differences in perceptions of when “old age” begins, as well as differences in experiences of age discrimination and the prevalence of intergenerational relationships (Abrams, Russell, Vauclair, & Swift, 2011). All of the studies in the current meta-analysis originate from either Europe or North America. Cultural, economic, social, and political differences between these continents may influence the experiences of older adults and may therefore alter the experience of ABST.

## Method

The present meta-analysis draws on 37 identified ABST studies to assess the strength of ABST effects as well as whether they are moderated by experimental differences and sample characteristics.

### Article Selection Criteria

General population evidence shows that older adults do self-categorize themselves according to age and are generally aware of stereotypes regarding their age group (e.g., Abrams et al., 2011). Following this premise, articles were selected based on meeting relevant criteria for stereotype threat (Steele, 2010). The first criterion for inclusion was the presence of an objective measure of performance. Studies were excluded if they did not include performance-based dependent variables (e.g., Auman, Bosworth, & Hess, 2005; Coudin & Alexopoulos, 2010; von Hippel, Kalokerinos & Henry, 2013). Second, studies must manipulate the relevance of the performance task to salient negative age-based stereotypes, in order to ensure that the performance setting is diagnostic of the age-based stereotype. When a stereotype is primed without the individuals’ awareness (e.g., subliminally or through other forms of priming such as sentence-unscrambling tasks), the stereotype may become more cognitively accessible and directly affect associated behavior (Bargh & Pietromonaco, 1982), this is stereotype priming. Studies were excluded if the performance domain was not under threat, this included studies that used stereotype-priming methods or combined these with manipulations of stereotype threat (e.g., Bensadon, 2010; Hess, Hinson, & Statham, 2004). For example, Hess, Hinson, and Statham (2004) used a sentence unscrambling task in which target words represented common beliefs about older adults (Experiment 1), and a lexical-decision task, which presented consciously perceivable prime words (Experiment 2). These procedures involve explicit stereotype priming but not necessarily stereotype threat because the activation of the stereotype does not necessarily elicit the threat of confirming the stereotype.

A third criterion was that studies had at least one baseline comparison condition so that the threat effect could be quantified. Studies were excluded if they used a nonexperimental design (e.g., Scholl & Sabat, 2008; von Hippel et al., 2005). All remaining studies used a between-participants design whereby older adults were randomly assigned to the control or experimental group. Finally, searches only included articles written or translated into English. Two studies that explored ABST on young participants (Hehman & Bugental, 2013; Moldoff, 2010) were excluded because there were too few to permit separate meta-analysis.

### Literature Search

First, online database searches were carried out using a database for “Abstracts in Social Gerontology,” also “PsycINFO,” and “PsycARTICLES.” Search one included the terms “stereotype threat*” or “stereotypic expectancies*” AND “age*” or “elderly*” or “young*” or “old*.” Search two included the terms “age stereotype*” AND “performance*”. Nineteen published articles met the requirements for the meta-analysis (of 914). An additional three studies were extracted from a thorough search of the references in these articles. Overall, 22 published articles met the inclusion criteria.

Second, efforts were made to identify unpublished ABST studies. This is a technique used to address publication bias (Frattaroli, 2006; Hawkins, Blanchard, Baldwin, & Fawcett, 2008). All primary authors of the already identified published ABST studies were contacted. A number of organizations were also contacted (and complied) with requests for unpublished data. This produced five unpublished pieces of research suitable for the meta-analysis (Cassidy & Persson, n.d.; Desrichard, n.d.; Horhota, n.d.; Lamont, 2011; Popham & Hess, 2013). Finally, using ProQuest Dissertations and Theses, all theses published internationally since 1990 were searched. Five unpublished studies were found that were not already included in the list of published articles (Cavanagh, 2011; Kominsky, 2003; Lambert, 2011; Rahhal, 1998; Stein, 2001). This search was terminated in February 2013. In sum, the search revealed 32 published and unpublished articles, including 37 experimental studies.

### Statistical Considerations

Eighty-two effect sizes were drawn from the 37 studies. Some studies included additional conditions or factors. Of these, four included two-by-two designs, manipulating ABST as one factor and then manipulating a second independent variable, such as regulatory focus or time pressure (Cavanagh, 2011; Fritzsche et al., 2009; Gaillard et al., 2011; Hess et al., 2009). It was not possible to determine which level of the second independent variable was more in line with the other reviewed studies. Therefore, ABST effect sizes were calculated separately at each level of the second independent variable (see Table 1). Relatedly, Abrams et al. (2008) included an imagined contact task to eliminate the effects of threat in a 3-level design (control, threat, threat + imagined contact). Only the first two conditions were used for the meta-analysis.[Table-anchor tbl1]

### Retrieval and Independence of Effect Sizes

Multiple effect sizes obtained from different studies within an article were considered to be statistically independent. However, multiple effect sizes were sometimes obtained from individual studies whereby both control and nullification conditions were included in comparison with the threat condition. Effect sizes sharing participants (*N*) due to the inclusion of both baseline conditions (control and nullification) were considered to be independent tests based on the distinct comparison that they form with the threat condition.

In addition, some experiments included more than one type of dependent variable, each yielding its own effect size. Although all studies with multiple dependent variables measured them within the same session, the sequence of measurement is a potential confound; for example, effects may become weaker if measured later rather than earlier. Also, in Horton et al.’s (2010) study the ABST manipulation was followed by measures of walking speed, physical self-description, and recall performance. After that, measures of reaction time (RT), grip strength, and flexibility were counterbalanced. To try to accommodate effects of differences in sequential measurement position of any particular dependent measure we conducted separate analyses for dependent measures that were recorded at different points (placements) in the sequence. Specifically, we distinguished effect sizes from measures that were either the sole dependent variable or that were taken earliest in a sequence after the ABST manipulation (first placement), and those that were from studies with multiple dependent variables and where the measure was either in the second, or in third or subsequent placements in the sequence. We recognize that even this approach does not account for situations in which a series of performance measures is also preceded by or interspersed with other measures, such as evaluation apprehension (Chasteen et al., 2005), expected performance (Desrichard & Kopetz, 2005) or an Implicit Association Test (Hess et al., 2003). The implications of the inclusion and placement of other types of dependent variable could be investigated once there is a larger set of studies available but is beyond the scope of the present analysis.

Effect size, *N*, and degrees-of-freedom (*df*) were obtained for all tests of ABST and are summarized in Table 1. Positive effect sizes indicate that the performance outcomes were in line with stereotype threat predictions whereby threat reduced performance. The standard mean difference between conditions (*d*) was computed from means and standard deviations or from alternative effect sizes (*t* and *F*). Authors were contacted for additional information where necessary.

### Coding Procedure

The first author coded sample characteristics (age and gender), dependent variable placement and whether the statistic was published in a European or North American journal. All other moderators were coded by the first and second authors and one independent coder, who were blind to the aims of the research. All variables coded showed high interrater agreement (Cohen’s kappa or κ; Cohen, 1960; Mean κ = .95). Any discrepancies in coding were discussed and final coding agreed upon.

#### Experimental manipulations of ABST

ABST manipulations that included a statement or evidence of factual difference in age-based performance were coded as *fact-based* (62%). All other manipulations were coded as *stereotype-based* (κ = .90). Manipulations in two studies highlighted differences in performance based on age but the information did not explicitly state that performance declines with age (Hess et al., 2009; Fritzsche et al., 2009). These were categorized as fact-based because of their use of statements of “factual differences.”

#### Stereotyped performance domains

We distinguished the following categories of performance domain: memory, cognitive, physical, skill acquisition, and driving*.* These are defined below. Studies were categorized into these domains by each coder (κ = .98; summarized in Table 1). The majority of studies focused on *memory* performance, which we defined as measures of recall, recognition and cued memory for novel words, sentences, shapes, and information (e.g., Chasteen et al., 2005; Kang & Chasteen, 2009; Cassidy & Persson, n.d.).

A second category of studies has focused on broader *cognitive* performance—tasks that require cognitive effort and other cognitive skills (Abrams et al., 2008; Abrams et al., 2006; Haslam et al., 2012; Horhota, n.d.; Hehman & Bugental, 2013; Popham & Hess, 2013; Swift, Abrams, & Marques, 2013). For example, these studies measured performance on math tests (Abrams et al., 2008), a letter cancellation task (Popham & Hess, 2013), a crossword task (Swift et al., 2013), a block design task (Hehman & Bugental, 2013), a mental rotation task (Horhota, n.d.) and other mixed tests of cognitive ability that sometimes include a memory component (Abrams et al., 2006; Haslam et al., 2012).

Additionally, two studies, classified as cognitive, tested a performance domain that was ambiguously linked to the stereotype they sought to manipulate (Desrichard & Kopetz, 2005; Gaillard et al., 2011). Desrichard and Kopetz (2005, Study 1) manipulated the presence of age-based memory stereotypes and sought to capture the effect on a “running an errand” task. Performance scores were based not just on memory but also the effectiveness of the strategy used. Gaillard, Desmette, and Keller (2011) manipulated the salience of both poor driving skill and reduced cognitive efficiency age-stereotypes, the effects of which were measured on multiple-choice questions about driving.

A third area of performance is *physical competence and motor skills*. Horton, Baker, Pearce, and Deakin (2010) measured walking speed, flexibility and RT and Swift, Lamont, and Abrams (2012; also Lamont, 2011) measured handgrip strength.

Some dependent measures could not readily be categorized within these three domains. Fritzsche, DeRouin, and Salas (2009) required participants to learn to use a new computer-based library cataloguing system, and then tested these new skills. This study targeted stereotypes of age-related differences in skill-acquisition (not memory), so the study was classified as *skill-acquisition*. Second, studies focused on stereotypes of older adults’ poor driving ability and driving performance (Joanisse et al., 2013; Lambert, 2011). These tasks required both cognitive and physical competence rather than just one performance domain. Therefore, these tasks were categorized as *driving*.

#### Baseline conditions

Baseline conditions were coded either as a *control* condition which did not mention age, or as a *nullification* condition that explicitly challenged the stereotype, for example by stating that the task is not age-biased or that no age differences have previously been found (44% of effect sizes; κ = .93; see Table 1 for categorization).

#### Age and gender

Where available, the percentage of participants that were female (*gender*) and the mean *age* of participants were recorded. Some studies gave only mean age/percentage female across conditions (e.g., across the threat, control, and nullification conditions), in these instances this best estimate of the mean/percentage was used.

#### Region of study

This was classified according to whether the study was conducted in *North America* or *Europe*. When region was unspecified in the Method section, it was classified according to the corresponding author’s location (κ = 1). *Journal region* was also classified based on whether the journal was based in *North America* or *Europe*.

### Meta-Analytic Procedure

Effect sizes (*d*), *N* and moderator values for each test of ABST were entered into SPSS (Version 18). The procedures and macros of Lipsey and Wilson (2001) were used to carry out transformations, meta-analyses, and moderator analyses. A random effects model—which takes into account both the between-study and within-study variance when weighting effect sizes—was used due to methodological heterogeneity between the studies (Mullen, 1989). Individual standardized mean difference scores were transformed to account for small sample size bias. Inverse variance weights, incorporating both within- and between-study variance (Tau-squared;τ^2^) were calculated to take into consideration the precision of individual effect sizes. These weights were then used to carry out inverse variance weighted meta-analyses (Hedges & Olkin, 1985; Lipsey & Wilson, 2001).

For each meta-analysis, a mean effect size was calculated, along with upper and lower 95% confidence intervals [CI_.95_ lower and CI_.95_ upper] and homogeneity of variance. Homogeneity of variance (Cochran’s *Q*) indicates whether effect sizes are significantly heterogeneous (beyond sampling error). Significant findings on this test would suggest that there are some real differences across studies that may be explained by moderator variables.

To model between study variability, mixed effects moderator analyses were conducted. For each categorical moderator a chi-square test is reported whereby *Q*_between_ and *p* show the size and significance of the variability in effect sizes between different levels of the moderator. For continuous moderators a meta-analytic regression was performed with all continuous variables entered into the same model. *Beta* and *p* show the predictive value of the moderators in explaining variance in effect sizes. For each categorical moderator, separate meta-analyses were conducted at each level of the moderator (see Table 4). Weighted mean *d* were interpreted according to Cohen’s (1988) criterion stating that small, medium and large effect sizes correspond to *d* = .2, *d* = .5, and *d* = .8, respectively.[Table-anchor tbl4]

## Results

### Age-Based Stereotype Threat Effects

The first meta-analysis was used to establish the overall effect of ABST on performance measures. A compilation of all 82 effect sizes was not possible due to crossover in *N* where effect sizes were derived from the measurement of more than one dependent variable in a study. Consequently, separate analyses were conducted for each placement of dependent measures following the ABST manipulation; first placement (P1), second placement (P2), and third placement and beyond (≥ P3). Table 2 presents all the effect sizes in stem-and-leaf plots for each placement.[Table-anchor tbl2]

The plot of P1 effect sizes shows a very broad spread of effect sizes (ranging from −4.4 to 5.5).^1^ The basic random effects analysis of all effect sizes at P1 (*k* = 53) supports the predictions of ABST theory, with a small to medium effect (mean *d* = .28). The effect was no longer significant at P2 (*k* = 18, mean *d* = .35) or ≥ P3 (*k* = 11, mean *d* = .18). These results reveal that the significance of the ABST effect depends on the placement of the dependent variable, with significant impact on performance measured directly after the manipulation, which reduces to nonsignificance on subsequent measures at P2 and > P3. However, the difference between P1 and P2 was not significant (*Q*_between_ (1) = .19, *p* = .66). This may be due to the significant heterogeneity in effect sizes. Homogeneity of variance statistics show there is significant heterogeneity among effect sizes for P1, P2, and ≥ P3, justifying the use of moderator analyses to explain variance in effect sizes (see Table 3). Given the larger number and the significance of ABST effects at P1 and to ensure independence of effect sizes, subsequent moderator analyses were only conducted on P1 effect sizes.[Table-anchor tbl3]

### Publication Bias

As identified by Rosenthal (1979), unattained unpublished studies may exist that represent a bias against the publication of nonsignificant results. Moderator analyses show that article status (published vs. not) significantly predicted variability in effect sizes (*Q*_between_ (1) = 6.73, *p* < .01). The effect size is significant in published research (mean *d* = .42), but not in unpublished research (mean *d* = −.03). This confirms that there is bias toward the publication of significant ABST results. Both published and unpublished work in this area has been included in all further analyses to better estimate the *true* effects.

**Figure 1 fig1:**
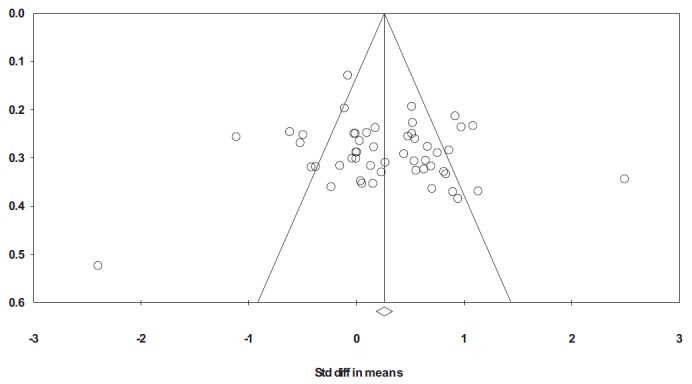
Funnel plot of P1 mean difference effect sizes (“Std diff in means”) plotted against the standard error of the mean difference effect sizes (“Standard Error”). Diagonal lines show 95% confidence limits and a vertical line shows the population effect size. Note: the two largest effect sizes (one positive and one negative) have not been included in the funnel plot due to their larger standard errors requiring a smaller display.

Further tests were conducted on the combined published and unpublished studies to identify whether it is likely that additional unpublished studies still exist. A nonsignificant correlation between effect size and sample size, *r*(51) = .04, *p* = .79, and a nonsignificant result using Eggers’s regression (β = 5.23, *p* = .36) suggest that our findings are not biased by the overrepresentation of smaller significant studies within the meta-analysis. A funnel plot (see Figure 1) also shows no obvious publication bias based on its symmetry around the population effect size. The plot shows that studies with lower standard errors (an indicator of precision) show less variability in effect sizes, as with an unbiased sample. Therefore, assuming we have a complete sample of unpublished studies, and given that these do not differ in sample size from the published studies, it seems reasonable to conclude that the most robust estimate of the overall meta-analytic effect size should include both published and unpublished studies.[Fig-anchor fig1]

### Moderators

#### Experimental manipulations of ABST

Stereotype-based manipulations revealed a significant mean *d* of .52. In contrast the mean *d* for fact-based manipulations was not significant (mean *d* = .09). Moderator analyses confirmed that threat manipulation type explained variation in observed effect sizes (*Q*_between_ (1) = 6.46, *p* < .05), demonstrating that stereotype-based threat manipulations produced significantly greater decrements in performance than fact-based manipulations (when contrasted with the baseline condition).

#### Stereotyped performance domains

Moderator analyses revealed significant variation in effect sizes based on performance domain (*Q*_between_ (4) = 45.28, *p* < .001). However, the number of effect sizes included in three of these categories was very small (physical *k* = 3; driving *k* = 2; skill acquisition *k* = 2) limiting our interpretation of these effects. Effect sizes for the two most commonly measured performance domains are significantly different (*Q*_between_ (1) = 8.10, *p* < .005). ABST effects are larger for cognitive tasks (mean *d* = .68) than for memory tasks (mean *d* = .21).

Despite the difference between memory and cognitive tasks, it seemed informative and statistically reasonable to contrast these with more motor/skill based tasks (physical, driving, skill acquisition). Moderator analyses revealed significant variation in effect sizes based on these two broad categories of performance domain (*Q*_between_ (1) = 5.98, *p* < .05). ABST effects are larger for cognitive and memory tasks (*d* = .36) than other tasks (mean *d* = −.46).

#### Baseline condition

Mean *d* for control effect sizes was significant at .39 and mean *d* for nullification effect sizes was not significant at .12. A moderator analysis showed that baseline condition could not explain variation in observed effect sizes (*Q*_between_ (1) = 2.11, *p* = .15), providing no support for the suggestion that nullifying versus ignoring stereotypes of aging might produce different effects.

#### Age and gender

Mean age and gender did not explain variance in *d* (β = −.05, *p* = .72 and β = .18, *p* = .21, respectively), contrary to hypotheses that the older and female participants would reveal larger ABST effects.

#### Region

The effect of region of the study was significant (*Q*_between_ (1) = 18.52, *p* < .001). Mean *d* for European ABST studies was significant (mean *d* = .72), but the mean *d* for those conducted in North America was not (mean *d* = −.06). We observed that 34% of the studies conducted in North America were stereotype-based, compared with 67% in Europe. Therefore, we explored the effect of study region separately for each manipulation type. The region of study moderated the effect size of stereotype-based manipulations (*Q*_between_ (1) = 11.26, *p* < .001). The effect size was greater in Europe (*k* = 10; mean *d* = .82; 95% CI [.57, .93]) than North America (*k* = 13; mean *d* = .13; 95% CI [−.18, .43]), but region was not a significant moderator of the effect size of fact-based manipulations (*Q*_between_ (1) = 2.15, *p* = .14).

#### Journal region

Journal region also accounted for variance in effect sizes (*Q*_between_ (1) = 11.40, *p* < .001). Mean *d* was significantly greater for articles published in European journals, (*k* = 5; mean *d* = .94; 95% CI [.35, 1]) than North American journals (*k* = 30; mean *d* = .30; 95% CI [.09, .49]). We also observed a higher proportion of stereotype-based studies published in European journals (67%) than in North American journals (40%). Therefore, we explored the effect of journal region separately for each manipulation type. Journal region significantly moderated the effect size for stereotype-based manipulations (*Q*_between_ (1) = 15.04, *p* < .001), but not fact-based manipulations (*Q*_between_ (1) = .41, *p* = .52).

Based on these findings we now focus on stereotype-based studies only (*k* = 23). Notably, all the published effect sizes from North American studies (*k* = 7) were published in North American journals. Six of the nine effect sizes obtained within Europe were also published in North American journals. We tested the possibility that regional differences might be because European journals may have required larger effect sizes to meet their publication threshold. It was found that journal region moderated effect sizes of studies conducted within Europe (*Q*_between_ (1) = 9.15, *p* < .005); European journals (*k* = 3; mean *d* = .99; 95% CI [.52, 1]), North American journals (*k* = 6; mean *d* = .65; 95% [.52, .75]). In contrast, region of study did not moderate effect sizes for studies published in North American journals, (*Q*_between_ (1) = 2.06, *p* = .15): European (*k* = 6; mean *d* = .65; 95% CI [.52, .75]), North American (*k* = 7; mean *d* = .49; 95% [.27, .67]). Thus, publication region predicts effect size magnitude because European journals published larger ABST effects of stereotype-based manipulations. This could reflect either a self-selecting author bias or bias in European journals’ publication criteria for larger effects.

## Discussion

The present article complements and extends previous stereotype threat evidence on gender and ethnicity by providing a more complete and accurate picture of stereotype threat effects on the third major stigmatized social category, age. It also complements Horton et al.’s (2008) stereotype priming meta-analysis.

Using evidence from 32 articles (10 of which are unpublished) that provided 82 effect sizes, the overall finding is that stereotype threat negatively affects older people’s performance. This effect is significant and robust, albeit small-to-medium (.28) and thus somewhat smaller than the .38 in Horton et al. (2008). The analyses also revealed that effect sizes (*d*) ranged from −4.43 to 5.52, indicating substantial heterogeneity across studies. An important contribution of the present analysis was therefore to explain why this heterogeneity exists, providing a number of new insights and raising further questions for ABST research and stereotype threat theory more broadly.

### Are Stereotypes More Damaging Than “Facts”?

Across performance domains stereotype-based manipulations caused greater performance decrements than fact-based manipulations. The damaging effects of stereotype-based manipulations have significant societal implications given the prevalence of age stereotyping (Abrams et al., 2011; Levy & Banaji, 2002). According to Cuddy, Norton, and Fiske (2005) older adults are stereotyped as warm but less competent than their younger counterparts, this results in a paternalistic form of prejudice characterized by feelings of pity, but also admiration. It seems unlikely that pity results in explicitly hostile actions toward older adults, but it is likely to result in increased helping, and also exclusion within competence-based settings. Because ageism toward older adults is widely accepted and endemic in subtle forms (Nelson, 2002), it may often be difficult to disambiguate the intention behind actions toward older adults. Older adults may be constantly bombarded with cues to age stereotyping at the hands of often well-intentioned individuals. Future research should explore how these stereotype cues (e.g., patronizing tones, offers of help, social exclusion, etc.) may impact the performance and future intentions of older adults.

Stone and McWhinnie (2008) suggest that effects of different threat manipulations may depend on the task domain. For instance, subtle manipulations, such as stereotype-based manipulations, create ambiguity about the presence of threat, which may negatively impact cognitive load and tasks reliant on working memory (Schmader et al., 2008). However, under some circumstances, if a threat is unambiguous it may induce a prevention focus which could lead to ineffective and disruptive performance strategies on tasks that rely on more automatic procedures (Beilock et al., 2006). Therefore, an important question for future research is whether more blatant and fact-based manipulations may have stronger effects in other performance domains that are less dependent on working memory (e.g., physical tasks).

### Stereotyped Performance Domains

We explored the possibility that ABST would impact performance domains differently based on the different skill sets required and the stereotypes targeted. There were significant effects of ABST on both memory and cognitive performance, with stronger ABST effects on the latter. This may be because ABST in the cognitive domain presents a greater stereotypic threat or it could be that the cognitive measures used are more sensitive or reliable than memory tasks, and thus reveal the threat effect more clearly. The overall implication of these findings, however, is that ABST effects can significantly reduce both cognitive and memory performance. One ramification is that ABST might cause misleadingly poor results in clinical assessments of cognitive impairment, or work-place assessments of adult learning. Although the effects of ABST on the combined physical performance, driving and skill acquisition tasks were nonsignificant, our interpretation of this finding is tentative given the mixed results from the few studies included.

### Baseline Conditions

ABST effects did not vary as a function of whether control and nullification conditions served as a baseline. However, it is notable that the direction of differences was counterintuitive—nullification having the weaker effect. At present, however, the evidence shows that creating a situation that is age stereotype “blind” (control) is at least as effective as one that directly confronts age stereotypes (nullification) as a way to minimize ABST.

### Age and Gender

Some previous research (Hess & Hinson, 2006; Hess et al., 2009) suggests that ABST would have stronger effects at the start than later in old age, due to the initially increased salience and significance of stereotypes of aging. Alternatively, the relevance of ABST might simply increase with age. Although both possibilities seem plausible, the present analysis is the first to test these possibilities meta-analytically, and revealed there were no effects of participant’s age. Moreover, although older women were expected to experience greater ABST effects due to their potential to identify with two negatively stereotyped “threatened” groups, there was no support for that hypothesis either. As discussed below, these “null” findings do not rule out the possibility that age and gender moderate ABST, but they confirm that such moderation does not arise within the age range of the available studies.

A number of other sample characteristics could plausibly affect ABST. For example, these might include level of education or the physical and psychological health of participants. Further, individual difference variables such as, psychological age, stigma consciousness (Hess et al., 2009; Kang & Chasteen, 2009), domain identification (Hess et al., 2009; Joanisse et al., 2013), and self-efficacy (Andreoletti & Lachman, 2004; Desrichard & Kopetz, 2005) may be relevant as moderators. Unfortunately, an insufficient number of available studies included measures of these variables to allow meaningful comparisons of effect sizes. Thus, further research is required to explore their implications.

### Regional Differences

ABST effect sizes were larger in studies conducted in Europe than for those conducted in North America. However, this difference in effect sizes based on study region was not apparent when looking at effect sizes published in North American journals only. ABST effect sizes from studies published in European journals were found to be larger than those published in North American journals. This fact remained when looking at studies conducted in Europe alone. Thus, either through authors’ self-selection or through editorial process there is a higher effect size threshold for ABST evidence published in European journals than in North America journals. It remains to be seen whether there is a true cultural difference in ABST. As yet, no direct comparison between these two geographical locations or others has been made within the same study. Yet it is known that age stereotyping does differ across cultures (Levy, Ashman, & Slade, 2009), and even within Europe (Abrams et al., 2011). Therefore, future research will need to address the question of regional and cultural differences in ABST more directly.

### Comparison of Stereotype Threats

The ABST effect of .28 in the current meta-analysis is in line with Nguyen and Ryan’s (2008) findings for gender (*d* = .21) and race/ethnicity (*d* = .32). Yet, Nadler and Clarke’s (2011) meta-analysis found larger effects among African Americans (*d* = .47), and Hispanic Americans (*d* = .58), as did Walton and Spencer (2009) when combining effect sizes for both women and ethnic minorities (*d* = .48). However, age and gender are not numerical minorities and they also cross-cut other category memberships. Therefore, lower average effect sizes may mask important variation due to other group memberships within levels of gender or age.

We found no effects of control versus nullification baselines on ABST, however, gender-based stereotype threat effects are greater when compared with nullification baselines, and ethnicity-based stereotype threat effects are greater when compared with control baselines (Nguyen & Ryan, 2008). These differences reinforce the point that differences in group characteristics can lead to differences in the experience of stereotype threat (Shapiro, 2011).

Three of the four previous stereotype threat meta-analyses discussed in this article included unpublished research (Nadler & Clarke, 2011; Nguyen & Ryan, 2008; Walton & Spencer, 2009). Given the publication bias revealed in the present meta-analysis—whereby inclusion of unpublished research reduced the ABST effect size from .42 to .28—it is important that future meta-analyses also include unpublished research and that when scientific methods are rigorously adhered to, both smaller and larger effects justify publication.

### Limitations and Recommendations for Future Research

Steele and Aronson (1995) refer to stereotype threat as a “threat in the air,” meaning a potential threat that may become apparent in any situation in which the stereotype is relevant. The studies included in the present meta-analysis all used an experimental design, testing performance within a controlled setting. Test-like situations may arise at consequential times in the lives of older adults, for example, within employment selection, further education, or the medical/care/support setting. Research should further consider the chronic effects of ABST. Over time older adults may become sensitized to cues that their cognitive and physical capabilities will be noticed and evaluated in many settings, such that they implicitly pose test-like conditions. For example, these may arise in the workplace, or when asked to look after grandchildren, or when taking part in group-based activities. An important question therefore is whether ABST affects older adults’ ability to “perform” outside of formal test-based settings. Some research has begun to recognize this need for testing ABST in more varied settings (von Hippel et al., 2013), and to explore a wider range of performance outcomes, for example, dependent behaviors (Coudin & Alexopoulos, 2010) and learning outcomes (Fritzsche et al., 2009). It is also possible that some older adults may avoid the negative experience of ABST by disengaging from important activities (Osborne, 1997; Carstensen, Isaacowitz, & Charles, 1999). This may have implications for health and wellbeing.

One limitation (sometimes a practical impossibility) of stereotype threat research is the difficulty of establishing mediating processes. Although this meta-analysis establishes a significant overall ABST effect, it is unclear whether the “threat” has been registered at a conscious level. Some ABST studies do include manipulation checks for “threat-based concerns” as an indication that stereotype threat has been experienced. These have included both implicit measures of stereotype activation (Chasteen et al., 2005; Hess et al., 2003; Thomas & Dubois, 2011) and self-report measures of ABST (Chasteen et al., 2005; Gaillard et al., 2011; Joanisse et al., 2013; Swift et al., 2013). Although, self-reported thoughts/feelings have the potential to reveal threat-based concerns, their absence does not necessarily demonstrate the absence of threat because individuals may not be fully aware of their cognitive or affective state, or willing to report on it. In addition, individuals may report threat concerns as a defensive attribution to explain their performance.

Implicit measures can potentially reveal an association between older adults and negativity (Chasteen et al., 2005; Hess et al., 2003; Thomas & Dubois, 2011), but these only test for the activation of negative age-based stereotypes, not the self-relevance of these stereotypes or the threat they present. Furthermore, there is an ambiguity surrounding the nature of “stereotype threat” and how this manifests as an individual concern (Shapiro & Neuberg, 2007). Without confirming the presence of threat-based concerns or other stereotype threat specific mechanisms (e.g., anxiety, reduced motivation, cognitive depletion), it is unclear whether stereotype threat effects are being experienced rather than direct stereotype priming (Bargh & Pietromonaco, 1982).

Exploration of interventions to ameliorate stereotype threat effects may be both practically beneficial and also illuminate its proposed mechanisms. For example, both intergenerational contact and imagined intergenerational contact have been found to moderate the effects of stereotype threat (Abrams et al., 2008, 2006). This appears to work through familiar mechanisms of intergroup contact (Pettigrew, 1998), which can counteract stereotypes and mean that intergroup comparisons do not give rise to anxiety. Future ABST research should focus on exploring these and other social processes that can off-set potential ABST in test contexts.

The present meta-analysis is limited to old-age based stereotype threat. Hehman and Bugental (2013) found that highlighting stereotypes of young people as less experienced and wise than older adults resulted in a “stereotype challenge” effect whereby younger adults (mean age = 18.72) performed better when confronted with these stereotypes. Additionally, unpublished research by Moldoff (2010) found no ABST on younger adults when making age (inexperience) salient during a test of political knowledge. These studies highlight that younger people’s stigmatized status is temporary unlike that of older adults (Garstka, Schmitt, Branscrombe, & Hummert, 2004) and raises the question of when, during the life course, ABST changes its focus and direction, suggesting a need to explore such changes across a wider age range and longitudinally.

Recent evidence suggests that stereotype threat (e.g., Seibt & Förster, 2004) and more specifically, ABST (Barber & Mather, 2013; Hess, Emery, & Queen, 2009; Popham & Hess, 2013) may elicit a “prevention” focus (Higgins, 1999), whereby the individual aims not to perform poorly (as opposed to striving to perform well). It seems possible that this effect may depend on how performance is measured. For example, under time constraint and based on hit rate, performance scores for free recall of word lists may be poorer for those with a prevention focus. In contrast, those with a prevention focus may do better if the testing context permits more time or opportunities for error correction. Consistent with this idea, tasks that draw on experience or knowledge appear to offer a basis for stereotype boost (performance enhancement) among older people (Swift et al., 2013).

## Conclusions

This article has provided the first comprehensive review, meta-analysis, and evaluation of ABST research. There is clear evidence that older adults’ memory (*d* = .21) and cognitive performance (*d* = .68) is negatively affected by threat from age stereotypes, and that these effects persist across gender and age groups (within later life). Moreover, we have established that vulnerability is greater when threat is induced by stereotypes (*d* = .52) rather than by facts (*d* = .09), and occurs reliably when the dependent variable is proximal to the manipulation. Surprisingly, ABST was reduced at least as much by simply not invoking stereotypes (control) as by directly informing people that stereotypes are incorrect (nullification). The analysis also revealed a publication bias (*d* = .42), and an intriguing regional difference between effect sizes published in European journals (*d* = .94) and North American (*d* = .30). Overall, this analysis helps to complete the picture of stereotype threat effects across the major social categories of gender, ethnicity, and now age. It also highlights that ABST is a significant problem confronting older people and that it will be valuable to explore ways to lift that burden within formal test settings. Further research is required to establish the extent of ABST, for example, in domains that require more versus less working memory, and in less studied performance domains such as physical strength or driving. The bias against publishing nonsignificant findings demonstrated through this meta-analysis highlights the importance of including unpublished research within future meta-analyses of stereotype threat.

## Figures and Tables

**Table 1 tbl1:** Summary of Age-Based Stereotype Threat (ABST) Studies Included in the Meta-Analysis

Study	Condition	DV placement	*N*	*d*	DV type	Comparison group	ABST manipulation type	Publication status
Abrams et al. (2008), Study 1		1	51	.755	Cognitive	Con	S-B	Published
Abrams et al. (2008), Study 2		1	84	1.086	Cognitive	Con	S-B	Published
Abrams et al. (2006)		1	97	.921	Cognitive	Con	S-B	Published
Andreoletti & Lachman (2004)		1	33	.043	Memory	Con	F-B	Published
		1	32	.055	Memory	Null	F-B	Published
		2	33	.150	Memory	Con	F-B	Published
		2	32	.088	Memory	Null	F-B	Published
		≥3	33	.221	Memory	Con	F-B	Published
		≥3	32	.082	Memory	Null	F-B	Published
Cassidy & Persson (n.d.)		1	44	−.036	Memory	Con	F-B	Unpublished
		1	44	.540	Memory	Null	F-B	Unpublished
		2	44	−.021	Memory	Con	F-B	Unpublished
		2	44	.869	Memory	Null	F-B	Unpublished
Cavanagh (2011)	No prompt	1	66	.519	Memory	Null	F-B	Unpublished
Cavanagh (2011)	Prompt	1	65	.100	Memory	Null	F-B	Unpublished
Chasteen et al. (2005), Study 1		1	40	.816	Memory	Con	S-B	Published
Chasteen et al. (2005), Study 2		1	39	.835	Memory	Con	S-B	Published
Chasteen et al. (2005), Study 3		1	40	.631	Memory	Con	S-B	Published
Desrichard & Kopetz (2005), Study 1		1	40	5.518	Cognitive	Con	S-B	Published
Desrichard & Kopetz (2005), Study 2		1	60	2.495	Memory	Con	S-B	Published
		2	60	1.970	Memory	Con	S-B	Published
		≥3	60	1.103	Memory	Con	S-B	Published
Desrichard (n.d.)		1	240	−.076	Memory	Con	S-B	Unpublished
Fritzsche et al. (2009)	Self-paced	1	25	−2.396	Skill-acquisition	Null	F-B	Published
	Self-paced	2	25	−1.743	Skill-acquisition	Null	F-B	Published
Fritzsche et al. (2009)	Timed	1	25	−4.429	Skill-acquisition	Null	F-B	Published
	Timed	2	25	−2.767	Skill-acquisition	Null	F-B	Published
Gaillard et al. (2011)	Prevention	1	30	.947	Cognitive	Null	F-B	Published
Gaillard et al. (2011)	Promotion	1	31	−.231	Cognitive	Null	F-B	Published
Haslam et al (2012)		1	34	1.134	Memory	Null	F-B	Published
Haslam et al (2012)		2	34	1.630	Cognitive	Null	F-B	Published
Haslam et al (2012)		3	34	1.116	Memory	Null	F-B	Published
Hehman & Bugental (2013)		1	54	.864	Cognitive	Null	S-B	Published
Hess et al. (2003)		1	32	.706	Memory	Con	F-B	Published
		1	32	.899	Memory	Null	F-B	Published
Hess, Emery, & Queen (2009)	Deadline	1	45	.646	Memory	Null	F-B	Published
Hess, Emery, & Queen (2009)	No deadline	1	37	.236	Memory	Null	F-B	Published
Hess & Hinson (2006)		1	71	.179	Memory	Null	F-B	Published
Hess, Hinson, & Hodges (2009)		1	103	−.107	Memory	Null	F-B	Published
Horhota (n.d.)		1	32	.158	Cognitive	Con	F-B	Unpublished
Horton et al. (2010)		1	64	−.018	Physical	Con	F-B	Published
		1	64	−.006	Physical	Null	F-B	Published
		2	64	−.167	Memory	Con	F-B	Published
		2	64	.077	Memory	Null	F-B	Published
		≥3	64	−.033	Physical	Con	F-B	Published
		≥3	64	.038	Physical	Null	F-B	Published
		≥3	64	−.207	Physical	Con	F-B	Published
		≥3	64	.040	Physical	Null	F-B	Published
		≥3	64	.207	Physical	Con	F-B	Published
		≥3	64	−.364	Physical	Null	F-B	Published
Joanisse et al. (2013)		1	61	.547	Driving	Con	F-B	Published
Kang & Chasteen (2009)		1	42	.696	Memory	Con	S-B	Published
		2	42	.168	Memory	Con	S-B	Published
		≥3	42	.193	Memory	Con	S-B	Published
Kominsky (2003)	Prospective* memory	1	40	.136	Memory	Con	F-B	Unpublished
	Prospective* memory	1	40	−.150	Memory	Null	F-B	Unpublished
Kominsky (2003)	Recall memory*	1	40	−.375	Memory	Con	F-B	Unpublished
	Recall memory*	1	40	−.416	Memory	Null	F-B	Unpublished
Lambert (2011)		1	39	.558	Driving	Con	F-B	Unpublished
		2	39	.478	Driving	Con	F-B	Unpublished
Lamont (2011)		1	44	.000	Cognitive	Con	S-B	Unpublished
		2	44	.000	Physical	Con	S-B	Unpublished
Mazerolle et al. (2012)		1	110	.517	Memory	Null	S-B	Published
		2	110	4.000	Memory	Null	S-B	Published
O’Brien & Hummert (2006)		1	57	.032	Memory	Con	F-B	Published
		1	57	−.518	Memory	Null	F-B	Published
		2	57	−.351	Memory	Con	F-B	Published
		2	57	−.336	Memory	Null	F-B	Published
Popham & Hess (2013)		1	63	.482	Cognitive	Null	F-B	Advance online publication
Rahhal (DV gender; 1998)		1	48	.444	Memory	Con	S-B	Unpublished
Rahhal (DV truth; 1998)		1	48	.000	Memory	Con	S-B	Unpublished
Rahhal et al. (2001), Study 1**		1	48	.008	Memory	Con	S-B	Published
Rahhal et al. (2001), Study 2		1	56	.166	Memory	Con	S-B	Published
Stein (2001)	Memory + evaluation	1	65	−.492	Memory	Con	S-B	Unpublished
Stein (2001)	Memory only	1	70	−1.113	Memory	Con	S-B	Unpublished
Stein (2001)	Memory + time pressure	1	69	−.615	Memory	Con	S-B	Unpublished
Swift, Abrams, & Marques (2013)		1	80	.525	Cognitive	Con	S-B	Published
		1	80	.977	Cognitive	Null	S-B	Published
		2	80	.984	Cognitive	Con	S-B	Published
		2	80	1.022	Cognitive	Null	S-B	Published
Swift, Lamont, & Abrams (2012)		1	55	.664	Physical	Con	S-B	Published
Thomas & Dubois (2011)***		1	42	.271	Memory	Con	F-B	Published
*Note*. Where a second independent variable was used or multiple types of threat manipulation, “Condition” uses the phrasing from individual manuscripts to refer to the level of the second independent variable or threat manipulation used to form the effect size. DV = dependent variable; Comparison group = control (Con) or nullification (Null) comparison condition; Manipulation type = stereotype-based (S-B) or fact-based (F-B) manipulation; *N* = study sample size; *d* = standard mean difference.								
* A test of prospective memory was embedded within the test of recall memory making the placement of these two “memory” tasks indistinguishable, they have therefore been distinguished within this “condition” section. ** For both Rahhal et al. (2001) studies, response accuracy to new items were not included when forming an overall accuracy score due to ceiling effects. As distraction condition was not of interest for the purposes of this meta-analysis, overall accuracy scores in each condition were computed based on the combination of accuracy scores for all critical items (both with and without distraction). *** In Thomas & Dubois, (2011) susceptibility to falsely remembering related lures is the point of interest. However, this meta-analysis is concerned with looking at the effects of ABST on overall memory performance and therefore, performance scores were computed based on the accuracy of responses to all items (related lures, studied words and unrelated words).								

**Table 2 tbl2:** Stem-and-Leaf Plot of all Effect Sizes (ds) for ABST by Dependent Variable Placement

First		Second		Third and greater	
Stem	Leaf	Stem	Leaf	Stem	Leaf
−4.4	3	−4.4		−4.4	
−2.7		−2.7	7	−2.7	
−2.4	0	−2.4		−2.4	
−1.7		−1.7	4	−1.7	
−1.1	1	−1.1		−1.1	
−.6	2	−.6		−.6	
−.5	2	−.5		−.5	
−.4	2 9	−.4		−.4	
−.3	8	−.3	5 3	−.3	6
−.2	3	−.2		−.2	1
−.1	0 1 5	−.1	7	−.1	
−.0	1 2 4	−.0	2	−.0	3
.0	0 0 1 3 4 5	.0	0 8 9	.0	4 4 8
.1	0 4 6 7 8	.1	5 7	.1	9
.2	4 7	.2		.2	1 2
.3		.3		.3	
.4	4 8	.4	8	.4	
.5	2 2 3 4 5 6	.5		.5	
.6	3 5 6	.6		.6	
.7	0 1 5	.7		.7	
.8	2 3 6	.8	7	.8	
.9	0 2 5 8	.9	8	.9	
1.0	9	1.0	2	1.0	
1.1	3	1.1		1.1	0 2
1.6		1.6	3	1.6	
1.7		1.7	0	1.7	
2.4	9	2.4		2.4	
4.0		4.0	0	4.0	
5.5	2	5.5		5.5	

**Table 3 tbl3:** Meta-Analytic Results by Dependent Variable Placement

			95% CI for *d*			
*d* inclusion criteria	*k*	*d*	Lower	Upper	τ^2^	*Q*^*a*^
Placement 1	53	.279**	.097	.443	.399	319.640***
Placement 2	18	.349	−.168	.715	1.228	244.347***
Placement 3	11	.184	−.077	.422	.118	25.207**
*Note*. *k* = number of effect sizes included; *d* = inverse variance weighted standard mean difference of meta-analyzed studies; = τ^2^ or between-studies variance; *Q* = homogeneity of variance (Cochran’s *Q*).						
^a^ For each *Q* test, *df* = *k* −1.						
* *p* < .05. ** *p* < .01. *** *p* < .001. One-tailed.						

**Table 4 tbl4:** Meta-Analytic Results for Hypothesized Moderators Using Only First Placement Effect Sizes

			95% CI for *d*			
*d* inclusion criteria	*k*	*d*	Lower	Upper	τ^2^	*Q*^*a*^
Published	35	.424***	.196	.609	.483	230.688***
Unpublished	18	−.029	−.233	.177	.124	47.376***
Fact-based manipulation	30	.086	−.123	.287	.239	105.382***
Stereotype-based manipulation	23	.520***	.248	.717	.542	202.783***
Memory	34	.210*	.020	.385	.242	140.252***
Cognitive	12	.681***	.399	.845	.420	68.733***
Physical	3	.193	−.221	.548	.071	4.124
Driving	2	—	—	—	—	—
Skill acquisition	2	—	—	—	—	—
Cognitive and memory	46	.355***	.179	.509	.342	248.967***
Other	7	−.462	−.860	.286	1	67.290***
Control comparison	31	.386**	.151	.580	.436	203.986***
Nullification comparison	22	.122	−.164	.388	.370	115.377***
Region of study, North America	38	.059	−.124	.237	.239	150.967***
Region of study, Europe	15	.723***	.484	.862	.480	99.516***
Journal region, North America	30	.300**	.087	.486	.290	136.036***
Journal region, Europe	5	.941*	.348	.996	2.336	71.653***
*Note*. *k* = number of effect sizes included; *d* = inverse variance weighted standard mean difference of meta-analyzed studies; = τ^2^ or between-studies variance; *Q* = homogeneity of variance (Cochran’s *Q*).						
^a^ For each *Q* test, *df* = *k* −1.						
* *p* < .05. ** *p* < .01. *** *p* < .001. One-tailed.						

## Footnotes

1 The meta-analyses were rerun with effect sizes at three standard deviations from the mean or more Windsorised and also removed. This had little effect on the overall meta-analytic effect size. For example, the effect size for P1 is .28 and when the two studies were Windsorised and deleted (one positive and one negative), this statistic did not change at two decimal places. At P2 all statistics were within three standard deviations of the mean. Further details are available on request from the first author.
