# Correction to Lamont, Swift, and Abrams (2015)

**DOI:** 10.1037/pag0000269

**Published:** 2018-08

In the article “A Review and Meta-Analysis of Age-Based Stereotype Threat: Negative Stereotypes, Not Facts, Do the Damage” by Ruth A. Lamont, Hannah J. Swift, and Dominic Abrams (*Psychology and Aging,* 2015, Vol. 30, No. 1, pp. 180–193. http://dx.doi.org/10.1037/a0038586), some of the effect sizes in the meta-analysis were mistakenly calculated based on standard error (*SE*), rather than standard deviation (*SD*). The authors identified this problem for three of the 32 studies in the analysis. In addition, *SE* was incorrectly used in one of the original publications (Desrichard & Kopetz, 2005), and amendments have been made based on this also.

**Table:** 

Reference (as in Table 1)	*N*	Placement (as in Table 1)	Used *d* (as in Table 1)	Altered *d*	Comment on effect size change
Fritzsche et al. (2009)	25	1 (self-paced)	−2.396	−.665	From *large* to *medium to large*
		2 (self-paced)	−1.743	−.484	From *large* to *medium*
		1 (timed)	−4.429	−.454	
		2 (timed)	−2.767	−.380	From *large* to *small to medium*
Mazerolle et al. (2012)	110	1	.517	.070	From *medium* to *negligible*
		2	4.000	.539	From *large* to *medium*
Stein (2001)	65	1 (memory and evaluation)	−.492	−.084	From *medium* to *negligible*
	70	1 (memory only)	−1.113	−.191	From *large* to *negligible*
	69	1 (memory and time pressure)	−.615	−.106	From *medium to large* to *negligible*
Desrichard & Kopetz (2005), Study 1	40	1	5.518	.900	Remains *large*

The recalculations have minimal impact on the meta-analysis conclusions, but effect sizes calculated throughout the article needed to be updated. The meta-analysis still revealed a small-to-medium effect of age-based stereotype threat (ABST; *d* = .32). Two conclusions have changed from the original moderator analyses. Journal region did not significantly moderate effect sizes of stereotype-based studies conducted within Europe (*Q*_between_ (1) = 2.17, *p* = .14). Thus, reassuringly, it cannot be concluded that publication region predicts effect size magnitude or that there are different expectations for effect sizes based on the journal region. Because this issue was slightly peripheral to the central questions for the analysis, the central conclusions of the article remain unaffected. Further, the meta-analysis now supports the authors’ initial hypothesis that gender would moderate ABST effects (β = .36, *p* < .05), whereby women may experience greater ABST effects. The online version of this article has been corrected.

